# The Effect of Lycopene Supplementation on Mood Status and Quality of Life in Infertile Men: A Randomized, Double-Blind, Placebo-Controlled Clinical Trial

**DOI:** 10.22074/ijfs.2020.5888

**Published:** 2020-02-25

**Authors:** Mehran Nouri, Mohammad Hossein Nasr-Esfahani, Mohammad Javad Tarrahi, Reza Amani

**Affiliations:** 1 Students’ Research Committee, School of Nutrition and Food Science, Isfahan University of Medical Sciences, Isfahan, Iran; 2 Department of Reproductive Biotechnology, Reproductive Biomedicine Research Centre, Royan Institute for Biotechnology, ACECR, Isfahan, Iran; 3 Isfahan Fertility and Infertility Center, Isfahan, Iran; 4 Department of Epidemiology and Biostatistics, School of Health, Isfahan University of Medical Sciences, Isfahan, Iran; 5 Department of Clinical Nutrition, School of Nutrition and Food Science, Food Security Research Center, Isfahan University of Medical Sciences, Isfahan, Iran

**Keywords:** Anxiety, Depression, Lycopene, Quality of Life, Stress

## Abstract

**Background:**

Infertility is a major worldwide problem which is caused by several factors such as environmental, physiological, and genetic conditions. Lycopene is considered to be one of the most important antioxidants that can contribute to reducing or preventing the psychological damage that leads to infertility. Thus, the aim of this study was to evaluate the effect of lycopene supplementation on depression, anxiety and stress scales and quality of life in infertile men.

**Materials and Methods:**

In this randomized clinical trial, 44 infertile men with oligozoospermia were randomly divided into the following two groups: the experimental group was supplemented with 25 mg lycopene, once per day for 12 weeks, and the control group received a placebo, for 12 weeks. Anthropometric and dietary data, physical activity, mood status, including depression, anxiety, stress, and quality of life scores were recorded pre- and post-intervention. Depression, anxiety and stress were assessed using a 21-item questionnaire (DASS-21) and quality of life was examined using the WHO 26-qustion questionnaire (WHOQOL).

**Results:**

The baseline age and body mass index (BMI) were not significantly different between the two groups (age: 31.89 ± 2.51 and 32.15 ± 2.16 years old for intervention and placebo, respectively; P=0.732 and BMI: 27.20 ± 1.68 and 26.53 ± 1.53; for intervention and placebo, respectively; P=0.206). There were no significant differences in depression, anxiety and stress values between the two groups; however, depression score significantly decreased in both groups compared to the baseline levels (P=0.028 and P=0.031). No significant differences were observed in four domains of quality of life, except for psychological domain that was improved in the lycopene group compared to the baseline values (P=0.049).

**Conclusion:**

Short term supplementation of lycopene had no effect on mood status and quality of life, except for psychological status in infertile men (Registration number: IRCT20171105037249N1).

## Introduction

Infertility is a disease of the reproductive system defined by the inability to achieve a clinical pregnancy after 12 months or more of regular unprotected sexual intercourse (1). The World Health Organization (WHO), as well as epidemiological studies estimated that the prevalence of infertility in the world is about 15%, and in developing countries, in one out of every four couples, infertility is detected (2, 3). Various psychological and environmental factors such as age, environmental and occupational pollution, ionizing radiation, heavy metals, toxic chemicals, inadequate nutrient intakes, change in lifestyle, exposure to toxin, oxidative stress, depression and anxiety contribute to spread of this disorder (4, 5). For decades, stressrelated illnesses like depression and anxiety, have risen (6); for instance, Maroufizadeh et al. (7) reported that prevalence of depression and anxiety scale was 33.0 and 49.6% in infertile participants, respectively. Furthermore, various studies showed that the quality of life in infertile people is significantly lowered (8); Valsangkar et al. (9), in a study on 106 women who referred to the infertility center, showed that women with infertility had compromised quality of life.

The relationship between mental status and infertility is complex as infertility is a risk factor for mental illness and, psychological distress, can also be a risk factor for infertility (10). For various reasons, infertility increases the stressful conditions that cause mental harm (11). Several studies showed that oxidative stress or reduction of antioxidant defense, as well as the reduction of antioxidant enzymes, can contribute to mood symptoms (6). Moreover, changes in neurological signals, as well as inhibition of neurogenesis, which reduce the secretion of hormones and affect the synthesis of testosterone, can contribute to male infertility (12).

Dietary antioxidants, mainly vitamins E and C and β-carotene, have an important role in preventing reactive oxygen species (ROS) production lipid peroxidation (LPO) and DNA damage (13, 14). Recent studies showed antioxidants have protective effects on depressive symptoms (15, 16). Lycopene as a fat-soluble aromatic carotenoid and one of the most important antioxidants to protect against free radicals, protects against infertility in men (6). Since lycopene can alter the levels of antioxidant enzymes by altering the levels of ROS, it can contribute to reducing or preventing the psychological damage that affects infertility (6, 17).

Previous studies showed that deficiencies in antioxidants levels are major causes of oxidative stress and affect the mood status; also, they found a relationship between the level of ROS, mood status, quality of life and fertility, suggesting that various factors can negatively affect spermatogenesis through increasing the levels of ROS, and alteration of the redox balance, which favors oxidants over antioxidants (18-20). Little work has been done to explore the role of antioxidants in ameliorating mood status and quality of life in infertile people. Thus, we sought to evaluate the effect of lycopene supplementation on mood (i.e. depression, anxiety, and stress) and quality of life in infertile men.

## Materials and Methods

### Subjects

This double-blind clinical trial was conducted in winter and spring of 2018 at Isfahan Fertility and Infertility Center. Initially, individuals who had a history of primary and secondary infertility, for at least 5 years, were selected. After a thorough examination, 44 infertile men met the inclusion criteria. The inclusion criteria included infertile men with a sperm count less than 20 million per milliliter, normal sperm <65%, volume <3.0 ml, and average motility <60%, aged between 25 and 45 years, and not receiving any other treatments. All patients were required to stop all prior medical treatments for a period of ≥12 weeks and to sign written consent form to enter the study. The exclusion criteria included having a history of all related disorders including testicular atrophy, urinary tract infection, testicular torsion, asthenospermia, azoospermia, genital trauma, inguinal and genital surgery or other genital diseases such as current genital inflammation and cryptorchidism, anatomical disorders for example meatal stenosis, or endocrinopathy, use of androgens or antiandrogens, previous hormonal therapy, or use of cytotoxic drugs, anticoagulants, immunosuppressants or any antioxidant supplements. Patients with physiological and psychiatric disorders that may affect sperm and sexual performance, drug abuse and body mass index (BMI) ≥30 kg/m^2^, were also excluded (21, 22). The study protocol was approved by the Medical Ethics Committee at the Isfahan University of Medical Sciences (IR.MUI.REC.1396.3.325) and registered under the code of IRCT20171105037249N1 in the clinical trials registry of Iran.

### Study design

At the beginning of the study, subjects were randomly assigned to the intervention group [that was supplemented with 25 mg lycopene (produced by 21st Century Company, USA) once per day for 12 weeks], or the control group [that received placebo (starch) for 12 weeks] and patients were advised to take the palacebo pills at lunch or dinner meal. We used the standard formula suggested for clinical trials by considering the study with type I error of 5% (α=0.05) and type II error of 20% (β=0.20) to calculate the sample size. For randomization group, the intervention 22 patients were assigned to code A, and for the placebo group 22 were assigned to code B, through the method of convenience sampling. Sample size was calculated based on sperm concentration (23). All patients and the clinician that prescribed the supplements were blind to the treatment. In order to guarantee blindness, lycopene and placebo were prepared in similar appearance.

Lifestyle information, medical history, demographic data, alcohol and tobacco use, and supplement intake were recorded for all participants. Body weight was measured (in minimal clothing), and body fat was determined by bioelectrical impedance analysis (BIA) method using the Omron BF-511 set. To measure height, a fixed non-stretchable tape was used in standing position. Then, BMI was calculated in kg/m^2^. The Physical activity level was assessed using the short form International Physical Activity Questionnaire (IPAQ) (24). After initial screening, dietary intakes of all patients were collected using a 3-day dietary record at the beginning and end of the study and we calculated nutrient composition of the portion size, and subsequently, energy, carbohydrate, protein, fat and lycopene intakes were obtained from food composition Tables provided by the United States Department of Agriculture sources.

### Assessment of depression, anxiety and stress

Depression, anxiety and stress were assessed using a 21-item Questionnaire (DASS-21) for all participants pre- and post-intervention. The Cronbach’s alpha coefficient was obtained to show the reliability of the questionnaire (0.84). Similar internal consistency coefficients were reported previously (25). Each item uses a four-point response scale ranging from 0 (did not apply to me at all) to 3 (applied to me very much or most of the time). For each 3 subscales, 7 questions are considered. Then, based on the score given for each question, the score of each parameter of depression, anxiety and stress was calculated.

### Assessment of quality of life

A 26-questions form of World Health Organization Quality of Life Questionnaire (WHOQOL) was applied. The Cronbach's alpha for all sample, non-clinical and clinical was 0.82, 0.84 and 0.82, respectively (26). It should be noted that questions 1 and 2 are used to measure the overall QoL, and 24 items encompass dimensions including social, psychological, environmental and physical issues. Environmental health was measured by 8 items, physical 7 items, psychological 6 items and social 3 items. There is no overall score for the WHOQOL and each domain is calculated by summation of their specific items. Individual’s perception of quality of life is measured by summing the total scores for each particular domain. All domain scores are scaled in a positive direction (higher score indicates higher QOL).

### Statistical analysis

All data are reported as mean ± standard deviation or frequency (%). The Kolmogorov-Smirnov test was used to evaluate the distribution of data. An independent samples t test was used to analyze the initial variables, dietary intake, mood status and quality of life between the two groups considering normal distribution of variables. A paired t test samples was used to compare the intragroup variables preand post-intervention. To control the confounding variables (energy and carbohydrate), a MANCOVA test was applied to determine the differences between the groups post-intervention. Statistical analysis was performed using SPSS software version 16 (SPSS Inc., Chicago, IL, USA). A P<0.05 was considered significant.

## Results

**Fig 1:**
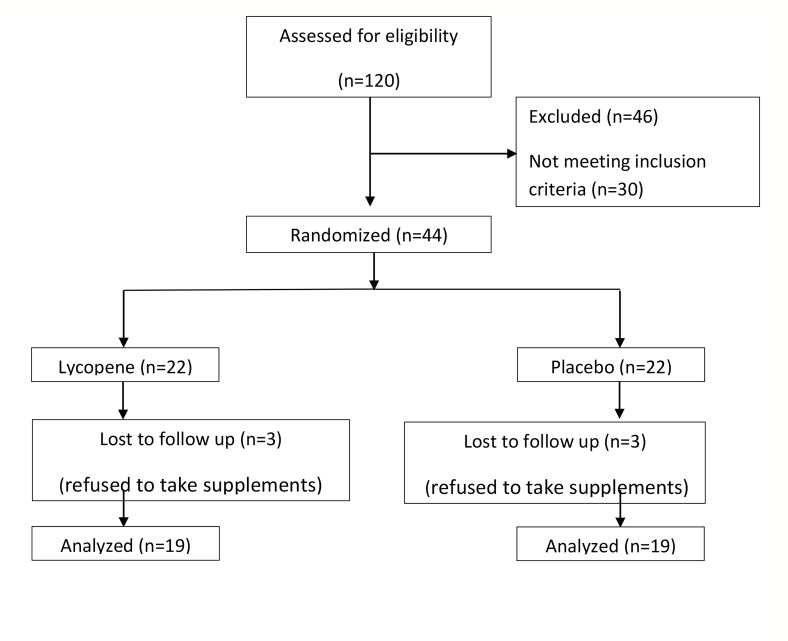
Flowchart of patient recruitment for the double-blind, placebo-controlled, randomized trial of lycopene supplementation in infertile men.

In total, 44 patients were recruited for this clinical trial and divided into two groups of 22 individuals; finally, 38 subjects completed the study: 19 in the lycopene group and 19 in the placebo group. In each group 3 participants refused to take supplements or participate in the final test, and thus, were removed from the study (Fig .1). Table 1 shows the basic characteristics and dietary intake of the patients. There were no significant differences regarding the baseline characteristics between the two groups, except for energy and carbohydrate intakes (P<0.05).

There were no significant differences in depression, anxiety and stress values between the two groups before and after adjustment of confounders using MANCOVA test (Table 2). Depression score decreased in both groups compared to the baseline values as assessed by pair t test (P=0.028 and P=0.031).

**Table 1:** Anthropometric and demographic characteristics and dietary intake of participants at baseline and end


Characteristic		Lycopene n=19	Placebon=19	P value

Age (Y)		31.89 ± 2.51	32.15 ± 2.16	0.732
Smoking history				0.740
	Yes	7 (36.84)	8 (42.1)	
	No	12 (63.16)	11 (57.9)	
Drinking alcohol history				0.721
	Yes	6 (31.57)	14 (73.69)	
	No	13 (68.43)		
Education				0.896
	≤12	3 (15.79)	4 (21.06)	
	High school diploma	7 (36.84)	6 (31.57)	
	Bachelor degree or higher	9 (47.37)	9 (47.37)	
Height (cm)		177.57 ± 4.79	178.78 ± 3.45	0.378
Weight (kg)		85.78 ± 6.10	84.78 ± 4.93	0.582
Body mass index (kg/m^2^)		27.20 ± 1.68	26.53 ± 1.53	0.206
Body fat (kg)		28.65 ± 3.37	27.98 ± 3.69	0.564
Physical activity (MET-h/week)		30.83 ± 1.95	31.0 ± 1.71	0.707
Energy intake (kilocalories/day)				
	Before	2251.39 ± 230.54	2115.53 ± 175.082	0.048
	After	2326.70 ± 200.01	2113.63 ± 199.87	0.002
Carbohydrate intake (g/d)				
	Before	316.56 ± 34.57	294.43 ± 28.0730	0.037
	After	330.41 ± 24.83	301.02 ± 27.96	0.002
Protein intake (g/d)				
	Before	88.04 ± 12.29	88.38 ± 8.52	0.922
	After	90.15 ± 12.65	86.11 ± 9.64	0.27
Fat intake (g/d)				
	Before	78.07 ± 16.35	72.61 ± 10.487	0.229
	After	79.59 ± 16.41	71.02 ± 11.11	0.068
Lycopene intake (µg/d)				
	Before	4306.46 ± 133	4664.39 ± 935.43	0.3450.885
	After	4895.57 ± 1362.35	4839.47 ± 961.29	


Data are presented as n (%) or mean ± SD. Analysis done using independent-sample t test.

**Table 2:** Depression, anxiety and stress score of participants at baseline and end


Variable		Lycopene n=19	Placebo n=19	P value^a^	P value^b^

Depression					
	Baseline	14.10 ± 2.94	13.78 ± 3.11	0.750	0.424
	End	12.73 ± 2.02	12.31 ± 2.13	0.537	
	P value^c^	0.028	0.031		
Anxiety					
	Baseline	11.26 ± 2.23	11.47 ± 3.04	0.809	0.510
	End	10.31 ± 2.13	10.84 ± 2.43	0.483	
	P value^c^	0.132	0.380		
Stress					
	Baseline	15.05 ± 2.34	14.52 ± 2.73	0.528	0.700
	End	14.52 ± 2.09	14.21 ± 1.98	0.636	
	P value^c^	0.331	0.546		


Data are reported as mean ± SD. a; Analysis done using Independent-sample t test, b; Multivariate analysis of covariance done following adjustment (for energy and carbohydrate), and c; Analysis done using paired-sample t test.

The effect of lycopene supplementation on four domains of quality of life (physical, psychological, social, and environmental) is presented in Table 3. There were no significant differences in all domains between the two groups before and after adjustment of confounders using MANCOVA test. Aside from the psychological domain in the lycopene group (P=0.049), no significant changes were observed in other quality of life domains as assessed by pair t test.

**Table 3:** Quality of life score of participants at baseline and end


Variable		Lycopene n=19	Placebo n=19	P value^a^	P value^b^

Physical health (%)					
	Baseline	67.73 ± 11.21	70.31 ± 17.017	0.585	0.743
	End	71.89 ± 10.20	2.89 ± 15.34	0.814	
	P value^c^	0.111	0.238		
Psychological health (%)					
	Baseline	66.36 ± 13.75	69.00 ± 19.39	0.632	0.998
	End	69.52 ± 10.99	71.57 ± 15.47	0.640	
	P value^c^	0.049	0.233		
Social relation health (%)					
	Baseline	72.05 ± 17.57	71.31 ± 22.28	0.911	0.680
	End	71.89 ± 12.16	72.89 ± 17.50	0.839	
	P value^c^	0.936	0.480		
Environmental health (%)					
	Baseline	67.36 ± 13.38	65.57 ± 21.45	0.760	0.578
	End	67.42 ± 11.25	65.52 ± 15.95	0.675	
	P value ^c^	0.977	0.980		


Data are reported as mean ± SD. ^a^; Analysis done using Independent-sample t test, ^b^; Multivariate analysis of covariance done following adjustment (for energy and carbohydrate), and ^c^; Analysis done using paired-sample t test.

## Discussion

This study was a randomized clinical trial designed to evaluate the effect of lycopene supplementations on depression, anxiety, stress, and quality of life. To the best of our knowledge this is the first study that assessed the effect of lycopene on mood state and quality of life scale. No significant differences were observed between the groups in terms of depression, anxiety and stress scores, or quality of life, after lycopene supplementation. Energy and carbohydrate intakes were different.

Our findings were in line with those reported by Tsuboi et al. (27) who assessed the correlations between serum lycopene and depressive score in 66 healthy female volunteers aged 38-70 years in 2000, and found no significant correlation between lycopene and depressive score. However, the results of other studies are equivocal. By conducting a cross-sectional study on 986 elderly Japanese individuals, Niu et al. found that a tomatorich diet is independently related to lower prevalence of depressive symptoms. However, they were ambivalent whether the protective effect of lycopene was directly caused by affecting the brain cells or by preventing depression-inducing diseases (6).

The antidepressant properties of lycopene were also observed in animal studies; for instance, Zhang et al. administered 6 mg/kg body weight per day lycopene for seven days to mice, and observed attenuated depressionlike behaviors (28). Moreover, Jain et al. (29) investigated the synergistic effect of a combination of lycopene, quercetin, and poloxamer 188 in a 3-nitropropionic acidinduced Huntington’s disease model, indicating that the combination of lycopene and quercetin is an effective nutritional component to alleviate and/or prevent the complications of Huntington’s disease, such as anxiety and depression.

Depression, anxiety, and stress are among the most prevalent mood disorders in the world (30). Since they can adversely affect the quality of life and are associated with infertility (31), the biological processes involved in the etiology of psychiatric disorders were studied. Oxidative stress is defined as an imbalance between cellular production of ROS and the counteracting antioxidant mechanisms (32). Since the brain consumes a high amount of oxygen and has a lipid-rich environment, it is highly vulnerable to oxidative stress (31). Also, due to the effects of smoking on oxidative status, and sperm quality, concentration, motility, and morphology, in this study, we recorded the history of smoking.

Besides, new studies point out that psychiatric disorders are resulted from alterations, not only in brain function, but also in neuronal plasticity (33). Increased free radicals could trigger such alterations, leading to cell death and atrophy of neuronal and glial cell population in the brain (34). Hence, powerful antioxidants, such as lycopene, are speculated to be protective agents against oxidative stress-induced neuronal damage since they are able to remove ROS. Lycopene could conceivably protect against this damage, resulting in the remission and functional recovery of depression or anxiety symptoms (35).

Another putative explanation for the potential protective effect of lycopene is based on its protective role against atherosclerotic cardiovascular diseases and cancer (36, 37). Since these chronic illnesses are also related to the occurrence of depressive symptoms.

The non-significant nature of our findings might be due to the relatively short duration and/or low dosage of administered lycopene, which might have been not high enough to exert stronger acute effects, highlighting the need for further work to investigate the impact of both duration and dosage. Nevertheless, contrary to contemporary theories, lycopene did not show any clinical effects on psychiatric disorders. It is noteworthy that within-group analysis showed that depression scores decreased in both groups compared to the baseline values. This could simply be due to the fact that the patients merely felt they are getting better while no clinical response to lycopene was evident. However, due to some constraints, we were unable to measure the seminal levels of lycopene, the receptors, and enzymes such as super oxidase dismutase (SOD) and catalase (CAT).

The authors of the present study strongly suggest that further work using varying doses, done in larger sample sizes, including both genders, and for longer periods should be conducted to evaluate the effects of potent antioxidants on different psychological aspects of infertile individuals.

## Conclusion

12-week lycopene supplementation, did not have any significant effects on psychiatric disorders and quality of life, urgently highlighting the need for further evidence of the efficacy of lycopene, for improving mood status and quality of life in infertile men.
